# Exploring for Municipality-Level Socioeconomic Variables Related to Zika Virus Incidence in Colombia

**DOI:** 10.3390/ijerph18041831

**Published:** 2021-02-13

**Authors:** Marie Kellemen, Jun Ye, Max J. Moreno-Madriñan

**Affiliations:** 1 Department of Global Health, Indiana University, Indianapolis, IN 46202, USA; mkelleme@iupui.edu; 2 Department of Statistics, University of Akron, Akron, OH 44325, USA; jye1@uakron.edu

**Keywords:** Zika, Colombia, socioeconomic, municipality, ecological study

## Abstract

Colombia experienced an outbreak of Zika virus infection during September 2015 until July 2016. This study aimed to identify the socioeconomic factors that at the municipality level correlate with this outbreak and therefore could have influenced its incidence. An analysis of publicly available, municipality-aggregated data related to eight potential explanatory socioeconomic variables was conducted. These variables are school dropout, low energy strata, social security system, savings capacity, tax, resources, investment, and debt. The response variable of interest in this study is the number of reported cases of Zika virus infection per people (projected) per square kilometer. Binomial regression models were performed. Results show that the best predictor variables of Zika virus occurrence, assuming an expected inverse relationship with socioeconomic status, are “school”, “energy”, and “savings”. Contrary to expectations, proxies of socioeconomic status such as “investment”, “tax”, and “resources” were associated with an increase in the occurrence of Zika virus infection, while no association was detected for “social security” and “debt”. Energy stratification, school dropout rate, and the percentage of the municipality’s income that is saved conformed to the hypothesized inverse relationship between socioeconomic standing and Zika occurrence. As such, this study suggests these factors should be considered in Zika risk modeling.

## 1. Introduction

The country of Colombia experienced an epidemic of Zika virus infection starting in September 2015 that lasted until July 2016 [1,2,3]. The time period analyzed in this study includes the outbreak time period and extends through the 12th epidemiological week of 2017. Zika is mainly transmitted by mosquitoes such as *Aedes aegypti* [4], which prefer urban environments and transmit other viruses such as dengue, yellow fever, and chikungunya [5,6]. The virus can be also transmitted in pregnancy from mother to child, sexually, and by blood transfusion [7]. Although symptoms of infection can include fever, rash, headache, or muscle pain [3] serious conditions such as the Guillain–Barré syndrome and particular danger to fetuses due to congenital abnormalities have also been linked to Zika virus infection [8].

Categories of Zika prevention/control include (a) individual-level protections against mosquitoes and (b) efforts to control mosquito populations. The Center for Disease Control and Prevention [9] website related to Zika virus emphasizes individual-level prevention such as self-protection from mosquito bites as the best way to prevent Zika. Weaver et al. [10] highlights the reduction in contact between the vector and susceptible humans as the best prospects for controlling Zika virus transmission, and points to the elimination or reduction in mosquito population as the most effective approach to reduce such contact. 

The opportunity for contact between mosquitos and humans and the resulting likelihood of pathogen transmission has been directly associated with a number of conditions. Among these are human population density [11]; poor lifestyle and housing conditions, such as the absence of air conditioning and screened windows [12,13]; and cultural practices, resulting from the lack of a continuous supply of piped water, such as water storage [14]. In turn, housing quality and water storage practices are largely dependent on socioeconomic conditions [15,16]. The absence of air conditioning and the presence of open non-screened windows lead to higher temperatures indoors (better habitat for mosquitoes) and more in-and-out access for mosquitoes, respectively, while water storage implies more opportunity for mosquito breeding sites [17,18]. The latter is an important sociocultural practice in response to the lack of piped water systems [11]. Indeed, next to climate-related conditions. (such as El Niño-Southern Oscillation (ENSO)), socioeconomic conditions are among the most important factors explaining outbreaks of vector-borne diseases. It has been suggested that socioeconomic conditions explain why *Aedes aegypti*-borne diseases are no longer prevalent in the United States but are still prevalent in developing American countries [15,16]. Studies conducted in Cali, Colombia, found high values of vulnerability to dengue virus in poor neighborhoods with high percentages of young, illiterate residents and high unemployment [19,20]. Similarly, socioeconomic factors helped explain the difference in the prevalence of dengue between sectors of the same city in Várzea Paulista, São Paulo, Brazil [21], and other health disparities [22,23,24].

Reducing the likelihood that an infected mosquito will bite a human reduces the number of infected mosquitoes and the opportunity of pathogen transmission. Whether transmission control takes the form of improving housing conditions, providing the continuous supply of piped water, or reducing the mosquito population, the application of such strategies depends on the financial capabilities and socioeconomic status of communities and their local governments. These are related variables [25], which in turn underly not only the mentioned determinants of health such as environmental exposure and health behavior, but also health care [22]. Therefore, the investigation of socioeconomic determinants of mosquito-borne diseases and in general of infectious diseases is of paramount importance [26]. The objective of this study is to identify area-level socioeconomic variables that are associated with the occurrence of Zika virus infection at the locality level in Colombia. As an exploratory analysis, this study’s ecological design is an appropriate approach for investigating the distal area-level factors (several of which have no individual-level analog) that may be associated with Zika virus infection in Colombia. This study aimed to identify new socioeconomic variables that could complement what has been reported in the literature [19,20], but also the set of physical environmental variables already used for mapping and modeling the incidence of dengue fever [27,28,29,30] and mosquito abundance in Colombia [31] and elsewhere [32]. 

## 2. Materials and Methods

### 2.1. Study Area 

**Figure 1:**
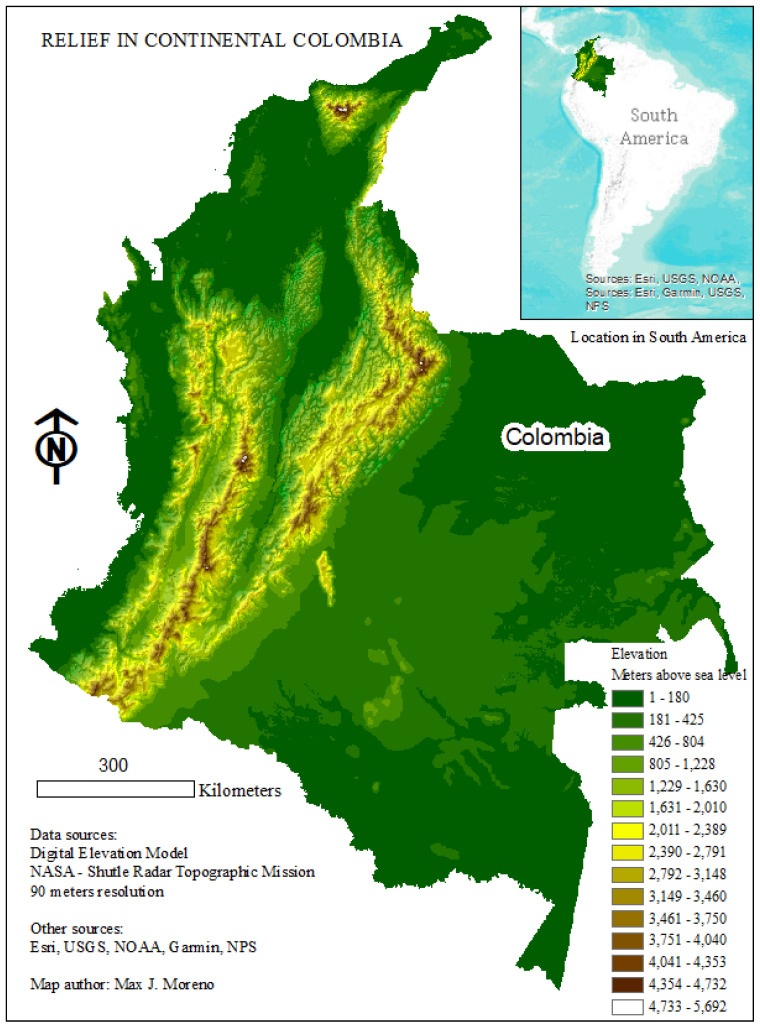
Map showing the elevation gradient of Colombia and its location in South America. Environmental System Research Institute (ESRI), United States Geological Survey (USGS), National Oceanographic and Atmospheric Administration (NOAA), United States National Park Services (NPS).

The study area for this analysis consisted of 934 localities within the mainland of the country of Colombia, which is located in the northwestern corner of South America (Figure 1). The geographical and political divisions of Colombia include (1) ‘departamentos,’ which are equivalent to states in the United States or Mexico, (2) ‘municipios, ‘distritos turisticos’ and ‘distritos turisticos culturales,’ which are equivalent to counties in the United States, are territorial entities at the level of sub-‘departamento’, and (3) other smaller localities within ‘municipios’ such as ‘corregimientos,’ This study examined 932 ‘municipios,’ one ‘distrito turistico,’ and one ‘distrito turistico cultural’. These three types of sub-departamental territorial entities are entitled to elect their own local ruler and count with their own rent, in addition to the economic support received from the central government. However, ‘distritos turisticos,’ and ‘distritos turisticos culturales’ have special characteristics such as tourist importance that differentiate them from regular ‘municipios’. For simplicity, the term municipality (English for municipio) will be used throughout the rest of this paper to describe all of the three types of territorial entities at the sub-‘departamento’ level examined herein. 

Colombia is an equatorial country located within the latitude range of the *Aedes aegypti* mosquito. Colombia counts with elevations higher than 5700 m above sea level, however, nearly 80% of the Colombian territory is located below 1800 m above sea level [19], which has been documented as the threshold for the survival of *Aedes aegypti* [33]. Colombia has a rich ecosystem diversity ranging from tropical rainforest to open savannas and deserts. Most of the country, 76.8% of the population (49.8 million people as of 2018) lives in urban areas [34] which *Aedes aegypti* prefers [35]. The fact that Zika was a new pathogen in the country, and thus there was no herd immunity, represents an opportunity to evaluate determinants without the confounding effect of prior exposures. These characteristics, in addition to the country’s very high income inequality [36], make Colombia an ideal site for this study. 

### 2.2. Variables

All data used in this study are publicly available, municipality-aggregated secondary data obtained from six Colombian governmental agencies: Instituto Nacional de Salud (INS, National Institute of Health of Colombia), Sistema Único de Información De Servicios Públicos Domiciliarios (Unique Information System of Residential Public Services, Bogotá, Colombia), Departamento Administrativo Nacional de Estadística (DANE, National Administrative Department of Statistics, Bogotá, Colombia), Instituto Geográfico Agustín Codazzi (IGAC, Agustín Codazzi Geographical Institute, Bogotá, Colombia), Ministerio de Educación (Ministry of Education, Bogotá, Colombia), and Ministerio de la Protección Social (Ministry of Social Protection, Bogotá, Colombia). Locality-identifying variables were used to merge the datasets containing the response and predictor variables together. This was performed using the statistical programming language R with version 3.5.1.

Epidemiological variables: although only one dependent variable was eventually selected to complete this analysis, two response variables were initially considered. These two variables were based on the total number of cases of Zika virus infection (including both suspected and confirmed cases) reported per municipality from the 32nd epidemiological week of 2015 until the 12th epidemiological week of 2017. The response variables were “ZikaDens” (eventually chosen) and “ZikaSize” (defined in Table 1). They were calculated from data on Zika cases downloaded from the website of the National Institute of Health (Instituto Nacional de Salud, INS) of Colombia [37]. Cases were processed according to INS protocols [38], meaning they were identified from patients’ symptoms and confirmed through laboratory tests, polymerase chain reaction and enzyme-linked immunosorbent assay, for patients with less and more than five days of symptoms, respectively. The epidemiological variable “ZikaDens” was calculated by dividing the number of reported cases of Zika virus infection by the weighted projected population density from the same time period. This is the number of cases per people per square kilometer (cases/people/km^2^). “ZikaSize” is the number of reported cases of Zika virus infection multiplied by 10,000 and divided by the weighted projected population size. This would be the number of cases per 10,000 people of the population (cases/10,000). Table 1 includes more details about how these variables were calculated. 

Two measures of Zika occurrence were initially considered to compare the outcomes with and without calculating population density within the dependent variable of Zika occurrence while using only socioeconomic explanatory variables. This choice reflects the expected importance of population density influencing the transmission of infectious diseases. Consequently, the analysis began by exploring whether “ZikaDens” or “ZikaSize” (for considering or not population density within the outcome variable, respectively) provides a better estimate of Zika occurrence when only considering socioeconomic explanatory variables. This limitation was imposed to prevent any overwhelming effect from a non-socioeconomic explanatory variable (such as population density) that could obscure the effect of any weak but still determinant socioeconomic variable. Subsequently, the different socioeconomic variables were analyzed for their significant influence on the chosen measure of Zika occurrence. 

Socioeconomic variables: the predictor variables used in this study were chosen from an internet search for publicly available data on variables that could potentially have an interest as socioeconomic indicators. The assumed criterion was that these variables could estimate socioeconomic status and vulnerability to Zika incidence. This search identified eight socioeconomic variables, for which data were available for the time period of 2014–2015, defined in Table 2.

Data on “school” were downloaded from the Ministry of Education (Ministerio de Educación) of Colombia [39]. This variable represents the school dropout rate of a municipio. Data on “low energy strata” were downloaded from the Single System of Information of Public Domiciliary Utilities (Sistema Único de Información De Servicios Públicos Domiciliarios) of Colombia [40,41,42,43,44]. Colombia’s energy supply utility weights its bills conforming to the socioeconomic stratum of the household, which is assigned according to the home’s physical characteristics and material conditions of its immediate environment [45]. These socioeconomic strata range from 1 (the lowest) to 6 (the highest) so the assigned stratum determines the amount residents pay for each unit of energy they use. For this analysis, we consider strata 1 to 3 to be “low energy” and calculate the variable as a weighted value for the percentage of households in a municipality who are in strata 1–3. Out of the total amount of subscribers (strata 1 to 6) during the 27 months of study period, only the percentage of residential subscribers in strata 1 to 3 during the full years 2015 and 2016 and three months of 2017 (which respectively correspond to the numbers 12 and 3 in the fractions of Table 2) were considered to calculate the variable of “low energy strata”.

The next chosen variable, “subsidized social sec” relies on the fact that Colombia has both a subsidized general social security system, with lower socioeconomic level enrollees, and a contributory social security system, with higher socioeconomic level enrollees. This is also a weighted value based on the percentage of people in a municipality who are enrolled in the subsidized system, based on data downloaded from the Ministry of Social Protection [46,47]. “subsidized social sec” and “low energy strata” are understood to represent lower socioeconomic status. The corresponding high socioeconomic version of each of these two variables were eliminated from the model to prevent a collinear effect between them. These three variables were clearly defined in the sources from where they were downloaded and easily understood as variables of potential socioeconomic importance.

The remaining five variables were less clear on their definitions, as well as on the interpretation as to whether they could accurately represent socioeconomic status. Data on variables coded as “tax” were downloaded from the Geographic Institute Agustín Codazzi (Instituto Geográfico Agustín Codazzi, IGAC) of Colombia [48,49]. This variable corresponds to the taxable base for the property tax for that municipality. Data from the National Department of Planning (Departamento Nacional de Planeación, Bogotá, Colombia) of Colombia [50] provides the “debt”, “savings”, “resources”, and “investment” variables for the municipal government [51,52,53]. The “debt” variable is the percentage of the municipality’s total revenue that corresponds to the total debt balance of that municipality. “Savings” is the percentage of the municipality’s income it saves. “Resources” is the percentage of the municipality’s total revenue, which is tax revenue. Lastly, “investments” is the percentage of the municipalities’ expenses that is invested. Table 2 provides more details on these variables.

### 2.3. Data Analysis

**Figure 2:**
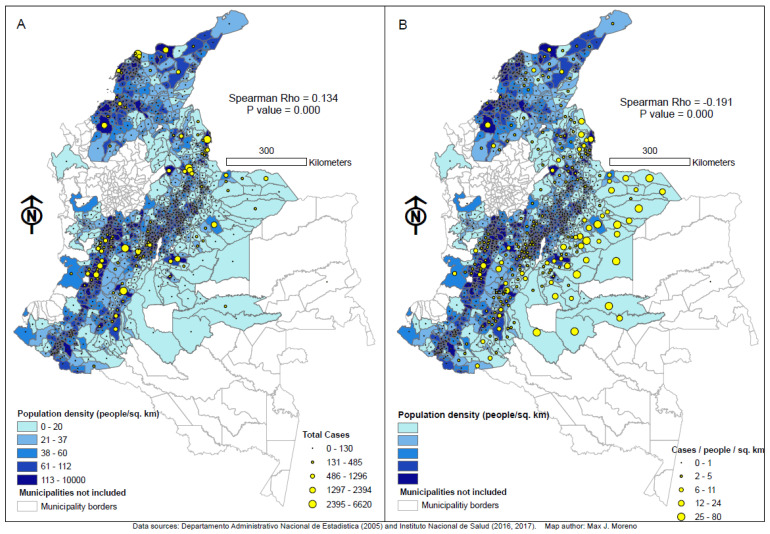
Visual comparison between the relationships of population density and reported cases of Zika virus infection (*r* = 0.134, (**A**)) and cases of Zika virus infection per people per square kilometer (*r* = −0.191, (**B**)) across 934 municipalities in Colombia from the 32nd epidemiological week of 2015 to the 12th epidemiological week of 2017. Population density is expressed as people/km^2^ in both choropleths (**A**,**B**). Reported cases of Zika virus infection are expressed as total cases in bubbles (**A**) while cases of Zika virus infection per people per square kilometer is expressed as cases/people/km^2^ in bubbles (**B**). The two variables depicted in (**A**) (choropleths and bubbles) are those used to calculate the variable cases of Zika virus infection per people per square kilometer (cases/people/km^2^). This resulting variable corresponds to bubbles in (**A**) and is the response variable “ZikaDens” described in Table 1A.

The independent variables were “school”, “low energy strata”, “subsidized social security”, “tax”, “debt”, “investment”, “savings”, and “resources”. The study’s response variable is based on counts of cases. A negative binomial model was selected from a list of four Poisson and four negative binomial regression models. The selection process was conducted by using Akaike’s Information Criterion (AIC) value [54] to compare between the models. The smaller the AIC value is, the better the model is (results of comparison are not included). Since the chosen negative binomial model has the smaller AIC value, we used it in the analysis of socioeconomic variables. Preliminarily, two different response variables, “ZikaDens” and “ZikaSize”, were considered. “ZikaDens” was chosen because its model has the smaller AIC value. In addition, “ZikaDens” controlled for population density (Figure 2A and Table 1A), allowing the comparison of exclusively socioeconomic variables as determinates. Therefore, as “ZikaDens” is the variable calculated with population density, its lower AIC was expected, given the important role of population density in dengue transmission, due to the increased opportunity for contact between humans and *A. aegypti* [11]. In sum, only the outcome from the selected binomial regression model with “ZikaDens” is discussed in extent here. Consequently, this chosen negative binomial model has the following form (see Table 1 for definitions of variable codes):

Log(ZikaDens) = b_0_ + b_1_(School) + b_2_(Tax) + b_3_(Low energy strata) + b_4_(Subsidized social Sec) + b_5_(Debt) + b_6_(Investment) + b_7_(Savings) + b_8_(Resources)

A comparison of the statistical significance and sign of the regression coefficients in the model enables conclusions to be drawn regarding how the relationship between Zika virus occurrence and the eight socioeconomic variables is affected. As mentioned earlier, all the above analyses were performed by statistical programming language R with version 3.5.1. Maps of the study area show population density, people per square km (people/km^2^) as choropleth (Figure 2A,B) as well as the number of Zika cases as yellow graduated symbols (Figure 2A). These two variables are the precursor variables used to calculate “ZikaDens”, cases per people per square kilometer (cases/people/km^2^), which is depicted in Figure 2B as yellow graduated symbols. These maps were designed with ArcMap 10.5 software and use the South America Albers Equal Area Conic projected coordinate system with a scale of 1:8,000,000.

## 3. Results

Descriptive statistics for the response and predictor variables are shown in Table 3. Data on the total number of cases of Zika virus infection in the study period are available for 1123 municipalities; one of these was missing information on population size and an additional 188 (17% of the total amount of municipalities in the country) were missing data on one or more predictor variables. Thus, only 934 municipalities were included in the regression models.

### 3.1. Geographical Distribution of the Occurrence of Zika Virus Infection across Colombia

Figure 2A depicts the relationships between population density (people per square km) and the total number of Zika cases. As explained earlier, these are the two precursor variables used to calculate the eventually chosen dependent variable “ZikaDens” (cases/people/km^2^), which is depicted in Figure 2B as yellow bubbles. The Spearman correlation between population density and Zika cases was 0.134 (Map 2A) while that between population density and Zika cases per people per square kilometer was −0.191 (Map 2B). Both relationships were highly significant (<0.001). As expected, the most populated areas (which correspond to the Andean and Caribbean regions, Figure 1) are those with the most cases (Figure 2A). Municipalities in the lowest quintile of population density generally have a lower occurrence of Zika virus infection. 

As expected, the map 2A suggests a positive relationship between population density and cases. Conversely, a first superficial look at map 2B may suggest an inverse relationship between population density and “ZikaDens” (cases/people/km^2^). However, such appreciation may be strongly influenced by the high records of cases/people/km^2^ in the municipalities located toward the east of the country. These are the eastern lowland areas known as Llanos Orientales (Eastern Planes, Figure 1), which account for 54% of the country’s area yet have less than 3% of the population [55]. They have much a lower coverage of public services as compared with the Andean and Caribbean regions. Thus, while the number of cases in the eastern side of the country (Figure 2A), is small, the numerator (Table 1A), population density in the denominator is even smaller as compared with municipalities in the west, creating a factor that increases the product of the equation. This explains the bigger yellow bubbles toward the east of the country and suggests other factors (socioeconomic and/or climatic) influencing Zika incidence besides population density.

### 3.2. Results of the Negative Binomial Regression Models

Table 4 shows the result of the selected negative binomial model. The response variable is ZikaDens. As shown in Table 4, six of the predictor variables have statistically significant regression coefficients. The regression coefficient for the predictor variables, school, low energy strata, taxes, investments, and resources were positive and statistically significantly related to ZikaDens. This indicates that the school dropout rate, the percentage of residential subscribers who are in the lowest strata for energy services, the taxable base for property, the percent of total expenses of the municipality that is invested, and the percent of the total revenue that correspond to taxes are directly statistically significant predictors of Zika occurrence.

Conversely, the variable for “savings”, which is the percent of current income per municipality that is saved, showed a significant inverse relationship with “ZikaDens”. The regression coefficients for the “subsidized social sec” (subsidized social security system) predictor variable and the “debt” predictor variable were not significantly related to “ZikaDens”.

## 4. Discussion

We believe that the reason for the relationships between Zika occurrence and the potential indicators analyzed here largely depends on the feasibility of the latter to represent socioeconomic conditions. Since the background section explains the possible relationship between socioeconomic conditions and Zika occurrence, much of the following discussion will be based on analyzing the possible feasibility of using these variables as indicators of Zika in terms of their feasibility as socioeconomic indicators. 

The positive sign of the “school” regression coefficient indicates that the rate of Zika virus infection increases with the increase in the school dropout rate. This finding is in line with the expectation that the occurrence of Zika virus infection increases as socioeconomic status decreases. School dropout and other related educational variables have long been associated with socioeconomic status [56]. In addition to the already explained high dropout-low socioeconomics relationship as an influencing factor for Zika, higher dropout rates may also influence disease occurrence due to the greater number of youths on the streets exposed to mosquito bites. The significance of the model for this variable supports the feasibility of using “school” (dropout rate) as an indicator of both socioeconomic condition and the likelihood of Zika occurrence. 

The results also show a significant regression coefficient with a positive sign for “low energy strata”. This suggests an increase in Zika virus infection when there is an increase in the proportion of people of low socioeconomic strata. A complementary version of this model was ran using the highest strata (strata 4 to 6 instead of strata 1 to 3) and the sign of the regression coefficient for this “high energy strata” was negative. This pattern is expected given the complementary nature of these variables, “low energy strata” and “high energy strata”, represent lower socioeconomic standing and higher socioeconomic status, respectively. As was the case for the school predictor variable, the sign of the energy predictor variable’s regression coefficients in our model (as well as in the complementary model) is in line with the assumption that the occurrence of Zika virus infection increases as socioeconomic status decreases. To the best of our knowledge, there has not been research exploring any association between mosquito-borne disease incidence and energy bill stratification. However, considering that in Colombia, the electricity billing rate is directly linked to the socioeconomic stratum in which the user is classified, which in turn is dependent on the physical characteristics of the home and material conditions of his immediate environment [44], this variable could be equivalent to housing quality. Several studies have found housing quality to be associated with dengue incidence [12,13,57] but one finds that it is not [58]. The housing conditions represented by the socioeconomic stratification used to determine the electricity-billing rate might be associated with a lack of mosquito-proof characteristics in housing, an abundance of containers suitable as breeding sites among other characteristics typical of lower socioeconomic conditions. These factors likely explain the relationship between Zika occurrence and energy strata. 

A third predictor variable in line with the findings from the “school” and “energy” variables, according to which lower socioeconomic status was related to higher Zika occurrence, is “savings”. We were not able to identify reports of association between “savings” and mosquito-borne disease in the literature. Although there is literature reporting evidence of a positive association between household precautionary savings and a variety of health outcomes [59] and insurance coverage [60] this reflects the fact that poor health can make it difficult for households to save rather than savings being the predictor of health. The present study found a negative and statistically significant regression coefficient between “savings” and Zika occurrence (ZikaDens). This finding suggests that more savings capacity in a municipio protects against Zika occurrence. This relationship, assuming a link between saving capacity and the socioeconomic standing, may indicate that a municipio able to save a greater proportion of its income enjoys a higher socioeconomic standing, which in turn results in lower Zika incidence. If the economic standing of municipalities relates to the economic standing of its inhabitants, the occurrence of Zika virus infection may increase as economic standing decreases. 

The variable “tax” corresponds to the taxable base for the property tax (Table 2). It is assumed that a higher taxable base would correspond to a higher socioeconomic level; therefore, the statistically significant and positive regression coefficient found for “tax” in the model seems to indicate an association between higher socioeconomic standing and higher levels of Zika occurrence. This unexpected result resembles others found in the literature. For example, Delmelle et al. [20] examined the relationship between socioeconomic status and rates of dengue fever in Cali, Colombia. They found that the regression coefficient for a socioeconomic variable in a geographically weighted regression model had a value near zero in some parts of the city but a value near +0.025 in other parts of the city. This may suggest the need for a weighted tax variable taking into account the high variability. If the socioeconomic–dengue occurrence relationship could vary on such a small scale (i.e., within a single city), then it seems logical that the relationship between various socioeconomic status or economic standing variables in this present study might have different relationships with Zika virus occurrence among municipalities. This unexpected positive relationship between the taxable base of property and Zika occurrence raises the question of whether the distribution of taxes across socioeconomic strata is appropriate and if there are possible confounding factors. Among these are the possibility that people in lower socioeconomic variables are reluctant to report cases. The lowest socioeconomic strata have a larger proportion of people with no steady employment such as hawkers, day laborers, etc., who may need to work even when they are sick. By contrast, those with steady formal employment have an added incentive to report their illness to health centers in order to explain their absence to their boss and are more prevalent in higher socioeconomic strata. 

The positive sign of the significant “resources” regression coefficient in the model indicates that as the ratio of a municipio’s tax revenue to total revenue increases, the occurrence of Zika virus infection increases. If a higher value of the “resources” predictor variable represents higher economic standing, then the regression results for this variable would, surprisingly, suggest that better economic standing is associated with more Zika virus infection. Greater awareness by the public, better public health infrastructure to detect cases, and therefore better case reporting in areas with more resources, may explain this result. Additionally, although more resources could lead to expect better mosquito control and Zika prevention practices, these could also be more focused in areas of lower resources, where the public perceive a greater risk due, for instance, to higher density of mosquitoes. Indeed, a study conducted in Malaysia found the highest odds of dengue prevention in lower income households [61]. However, because the source of non-tax revenue was not specified in the dataset obtained from the SIGOT tool (IGAC, n.d.) the degree to which the “resources” predictor variable indicates economic standing is unclear, as explained below.

The SIGOT (Spanish acronym for geographical information system for planning and order) tool (IGAC, n.d.) refers to the “resources” variable as the “generation of own resources (fiscal effort)”. This description seems to imply that tax revenue represents resources that the municipio generates for itself and its denominator includes that revenue from other sources, perhaps money obtained from the departamento or central government. It is also possible that some municipalities generate their own resources through some process other than taxation. If such income is not classified as “tax revenue” but is included in the “total revenue” denominator of the “resources” variable, then a municipio could have a low value of “resources”, yet a strong economic standing. Therefore, it is possible that the direct relationship between the “resources” predictor variable and the occurrence of Zika virus in the models might not indicate that higher economic standing is related to greater Zika occurrence. Similarly, resources from the departamento or central government may be lower than what they should be for poor municipalities, making the ratio of tax revenue to total revenue appear higher. Furthermore, as explained before for the “taxes” variable, another possible contributing confounding factor could be an inappropriate distribution of taxes. 

If the “investment” predictor variable (as estimated by the percentage of total investments to total expenses) represents higher economic standing, then the significance and positive sign of the regression coefficient for this variable in the model would suggest that better economic standing is associated with more Zika virus infection. An association between municipality investment and population density may explain this apparent contradiction. Indeed, municipalities located in the Caribbean and Andean regions (Figure 1), which are the most densely populated regions in the country (with about 65% of the country population [62]), are also those receiving more investment [63]. Population density may be associated with crowding, which may facilitate transmission, resulting in an association between the likelihood of virus transmission and investment. In addition, because the SIGOT tool does not specify the types of expenditures that are classified as investments (IGAC, n.d.) the degree to which the “investment” predictor variable indicates economic standing is unclear. For instance, if a municipality expends money to increase the quality of education or to increase the quality of physical infrastructure but these expenditures are not considered investments, then that municipality could have a lower value of “investment” without having a weak economic standing. Since there is no clarity whether the “investment” predictor variable reliably represents economic standing, the use of this variable as a predictor of Zika occurrence is not recommended. 

The lack of significance for “debt” and “subsidized social sec” means that no significant relationship was detected between these variables and Zika virus occurrence. To double check the effect of social security, instead of using the percentage of people enrolled in a subsidized general social security system, who are therefore assumed to be of lower socioeconomic level, a complementary model was run but using the percentage of people enrolled in a contributory social security system, who are therefore assumed to have higher socioeconomic level. This complementary model was not statistically significant as a response variable, either. This inability to detect a relationship between Zika occurrence and a variable that had been assumed to clearly represent socioeconomic status such as “subsidized social sec” can be explained again by a confounding effect of reporting misrepresentation. As a larger proportion of people on the subsidized social security system do not count steady jobs, they are consequently less likely to report illness compared to employees in a more formal economy, who are more encouraged to report illness to claim work-free days and who frequently count with a higher socioeconomic level. 

Ecological analyses such as this, using small area level data on disease linked to publicly available data on potentially determinant socioeconomic or environmental variables can be a powerful and efficient approach to estimate disease vulnerability. Therefore, important for public health preparedness for infectious diseases in general. Indeed, associations with socioeconomic-related variables such as ethnic concentration, residential instability, material deprivation, and income are being noticed for COVID-19 detection [64].

### Study Limitations

The population data for years 2015, 2016, and 2017 obtained from DANE [34] are projected based on values measured in the 2005 census conducted in Colombia. Because these projected population values were used in the calculation of both of the outcome variables in this study, discrepancies in the actual population values in 2015, 2016, and/or 2017 versus the projected population values would lead to inaccurate values for “ZikaDens” and “ZikaSize”. In addition, as mentioned earlier, the possibility of underreporting among people of lower socioeconomic status as they do not need a medical excuse for work free days, and are more likely to work when sick, may be a confounding factor.

For the discussion of the internal and external validity of the current study, definitions of internal validity will include those in Steckler and McLeroy’s discussion [65] of three works: Campbell and Stanley [66] and Cook and Campbell [67]. It is “whether or not observed covariation should be interpreted as a causal relationship” (p. 9) Appendix A. The relationships between the outcome and predictor variables that were tested in this study were not necessarily expected to be causal, but rather, potential indicators of Zika virus infection at the locality level.

## 5. Conclusions

The relationship between socioeconomic status and Zika virus occurrence is not consistent across all tested possible indicators of socioeconomic status or economic standing. The predictor variables of “school”, “low energy strata” and “savings” support the expected inverse relationship between socioeconomic status (or economic standing) and Zika virus occurrence, but not the five remaining variables. Out of these five, “subsidized social sec” and “debt” were not found to be significantly related to Zika abundance. “Tax”, “investments”, and “resources” (the later including a measure of tax for its calculation) suggested a direct relationship between higher economic standing and Zika virus occurrence, the opposite of the expected relationship, which may be due to confounding factors. 

A practical application for public health practice can be drawn from this preliminary work by considering the sign of the regression coefficients in the three variables found to be significant. Energy stratification, school dropout rate, and the percentage of the municipality’s income that is saved conformed to the hypothesized inverse relationship between socioeconomic standing and Zika occurrence. Furthermore, two of these three variables (energy stratification and school dropout rate) were clearly defined in the online sources where their data were downloaded and easily understood as variables of socioeconomic importance. As such, this study suggests they should be included in Zika risk modeling. As examples of possible public health applications, these variables could be used to identify municipalities and areas within municipalities where prevention efforts should be intensified based on the following criteria of (a) higher school dropout rates, a (b) higher percentage of individuals who are in strata 1–3 for energy services (lowest socioeconomic strata), and/or (c) municipalities with lower savings capacity.

## Figures and Tables

**Table 1:** Definitions of the preliminarily considered response variables.

Code	Definition
**A**	$ZikaDens=\frac{\left(\begin{array}{c} Number\;of\;reported\;cases\;of\;Zika\;virus\;infection\\ from\;the\;32nd\;epidemiological\;week\;of\;2015\;\\ through\;the\;12th\;epidemiological\;week\;of\;2017 \end{array}\right)}{Weighted\;projected\;population\;density\;for\;2015-2017}$
ZikaDens *(response variable 1)*	Weighted projected population density for 2015−2017= $\frac{\left(\begin{array}{c} \left(\frac{32}{96}\right)\times Projected\;municipio\;population\;in\;2015+\\ \left(\frac{52}{96}\right)\times Projected\;municipio\;population\;in\;2016+\\ \left(\frac{12}{96}\right)\times Projected\;municipio\;population\;in\;2017 \end{array}\right)}{Municipio\;area\;in\;{\mathrm{km}}^{2}}$
**B**	$ZikaSize=\frac{\left(\begin{array}{c} Number\;of\;reported\;cases\;of\;Zika\;virus\;infection\\ from\;the\;32nd\;epidemiological\;week\;of\;2015\;\\ through\;the\;12th\;epidemiological\;week\;of\;2017 \end{array}\right)}{Weighted\;projected\;population\;density\;for\;2015-2017}\times \mathrm{10},\mathrm{000}$
ZikaSize *(response variable 2)*	Weighted projected population size for 2015−2017= $\left(\frac{32}{96}\right)\times Projected\;municipio\;population\;in\;2015+$ $\left(\frac{52}{96}\right)\times Projected\;municipio\;population\;in\;2016+$ $\left(\frac{12}{96}\right)\times Projected\;municipio\;population\;in\;2017\;\;\;$

**Table 2:** Definitions of the predictor variables.

Code	Definition
School	$School=\frac{\begin{array}{c} Number\;of\;students\;enrolled\;at\;the\;beginning\;of\;the\;\\ school\;year\;in\;the\;levels\;of\;“primary”\;and\;“middle” \end{array}}{Number\;of\;students\;ending\;the\;year}\times 100$
Low energy strata ^1^	Energy= $\left(\frac{12}{27}\right)\times \left(\frac{\#\;of\;residential\;subscribers\;for\;energy\;services\;in\;strata\;1-3\;for\;2015}{\#\;of\;residential\;subscribers\;for\;energy\;services\;in\;strata\;1-6\;for\;2015}\right)\times 100+$ $\left(\frac{12}{27}\right)\times \left(\frac{\#\;of\;residential\;subscribers\;for\;energy\;services\;in\;strata\;1-3\;for\;2016}{\#\;of\;residential\;subscribers\;for\;energy\;services\;in\;strata\;1-6\;for\;2016}\right)\times 100+$ $\left(\frac{3}{27}\right)\times \left(\frac{\#\;of\;residential\;subscribers\;for\;energy\;services\;in\;strata\;1-3\;for\;Jan.-March\;2017}{\#\;of\;residential\;subscribers\;for\;energy\;services\;in\;strata\;1-6\;for\;Jan.-March\;2017}\right)\times 100$
Subsidized social sec	Social Sec A is % of people enrolled in some capacity to the general social security system who are enrolled in a subsidized way
Savings	$Savings=\frac{Current\;savings}{Current\;income}\times 100$
Tax	$\frac{\begin{array}{c} \left(Taxable\;base\;for\;the\;property\;tax\;in\;thousands\;of\;pesos\;in\;2015\right)+\\ \left(Taxable\;base\;for\;the\;property\;tax\;in\;thousands\;of\;pesos\;in\;2016\right)\;\;\;\;\; \end{array}}{2}$
Resources	$Resources=\frac{Tax\;revenue}{Total\;revenue}\times 100$
Investment	$Investment=\frac{Total\;investment}{Total\;expenses}\times 100$
Debt	$Debt=\frac{Total\;debt\;balance}{Total\;revenues}\times 100$

**Table 3:** Descriptives of the response and predictor variables.

	B	
	Among N = 934 Municipalities Used in Regression Analyses	
Response variable	Median	SD
ZikaDens	0.14	5.01
ZikaSize	5.62	41.11
Predictor variable	Median	SD
School	2.63	1.88
Low energy strata	99.95	4.24
Subsidized social sec	93.33	17.92
Savings	46.85	15.67
Tax	69,189,094	2,224,129,800
Resources	54.62	20.80
Investment	90.10	5.27
Debt	2.29	3.55

**Table 4:** Results of model: negative binomial regression ZikaDens as the outcome variable and metrics for goodness of fit.

	Estimate	Std. Error	z Value	Pr (>│z│	2.5%	97%	Signif. Codes.
Intercept	−1.12 × 10	1.973	−5.686	1.30 × 10^−8^	−1.51 × 10	−7.25	***
School	2.24 × 10^−1^	3.08 × 10^−2^	7.285	3.22 × 10^−13^	1.57 × 10^−1^	2.93 × 10^−1^	***
Low energy	4.72 × 10^−2^	1.77 × 10^−2^	2.659	0.007	1.06 × 10^−2^	8.34 × 10^−2^	**
Sub social sec	−5.41 × 10^−4^	4.91 × 10^−3^	−0.110	0.912	−1.18 × 10^−2^	1.06 × 10^−2^	
Tax	7.69 × 10^−11^	2.78 × 10^−11^	2.769	0.005	1.31 × 10^−12^	1.67 × 10^−10^	**
Debt	−9.12 × 10^−3^	1.66 × 10^−2^	−0.548	0.583	−4.26 × 10^−2^	2.58 × 10^−2^	
Investment	4.78 × 10^−2^	1.61 × 10^−2^	2.958	0.003	1.41 × 10^−2^	8.16 × 10^−2^	**
Savings	−1.53 × 10^−2^	4.43 × 10^−3^	−3.455	0.000	−2.52 × 10^−2^	−5.54 × 10^−3^	***
Resources	4.12 × 10^−2^	4.07 × 10^−3^	10.127	<2 × 10^−16^	3.28 × 10^−2^	4.98 × 10^−2^	***
Signif. Codes: 0 ‘***’ 0.001 ‘**’ 0.01 ‘.’ 0.1 ‘ ’ 1							
Deviance Residuals: Min: −1.7927, 1Q: −1.0168, Median: −0.8259, 3Q: −0.4452, Max: 4.6564							
Dispersion parameter for negative binomial (0.4952) family taken to be 1							
Null deviance: 1266.8 on 933 degrees of freedom							
Residual deviance: 963.2 on 925 degrees of freedom							
Number of Fisher scoring iterations: 1							
AIC (smaller is better): 2593.237							

## Data Availability

All data used in this study are publicly available, municipality-aggregated secondary data obtained from seven Colombian governmental agencies: Instituto Nacional de Salud, Sistema Único de Información De Servicios Públicos Domiciliarios, Departamento Administrativo Nacional de Estadística (DANE), Instituto Geográfico Agustín Codazzi (IGAC), Ministerio de Educación, and Ministerio de la Protección Social. Health data were downloaded from the website of the National Institute of Health (Instituto Nacional de Salud, INS) of Colombia (2016, 2017). They were calculated from data on Zika cases downloaded from the website of the National Institute of Health (Instituto Nacional de Salud, INS) of Colombia (2016, 2017). Instituto Nacional de Salud (INS) (2016). *Casos Zika por municipio semana 52 2016* (data file). Retrieved from http://www.ins.gov.co/Noticias/Paginas/Zika.aspx#.WXpN5IjyvIX. Instituto Nacional de Salud (INS) (2017). *Vigilancia rutinaria por evento_departamento_municipio periodo_III* (data file). Retrieved from: http://www.ins.gov.co/Direcciones/Vigilancia/Paginas/SIVIGILA.aspx. The percentage of people in a muncipio who are enrolled in the subsidized system, based on data downloaded from the Ministry of Social Protection (Ministerio de la Protección Social, 2014a, b). Ministerio de la Protección Social. (2014a). *Afiliados al régimen contributivo* (data file). Retrieved from: http://sigotn.igac.gov.co/sigotn/reporteMapaNuevo.aspx?prmAlls=221;11;2014;1;0;0;655674;Afiliados%20al%20R%C3%A9gimen%20Contributivo; Ministerio de la Protección Social. (2014b). *Afiliados al régimen subsidiado* (data file). Retrieved from http://sigotn.igac.gov.co/sigotn/reporteMapaNuevo.aspx?prmAlls=102;11;2014;1;0;0;655674;Afiliados%20al%20R%C3%A9gimen%20Subsidiado; Data on “school” were downloaded from the Ministry of Education (Ministerio de Educación) of Colombia (2015). Ministerio de Educación. (2015). *Tasa de deserción escolar total* (data file). Retrieved from: http://sigotn.igac.gov.co/sigotn/reporteMapaNuevo.aspx?prmAlls=114;10;2015;1;0;0;655674;Tasa%20de%20Deserci%C3%B3n%20Escolar%20Total; Data on variables coded as “tax”, were downloaded from the Geographic Institute Agustín Codazzi (Instituto Geográfico Agustín Codazzi, IGAC) of Colombia (2015, 2016). Instituto Geográfico Agustín Codazzi (IGAC) (n.d.). *SIG-OT*. Retrieved from: http://sigotn.igac.gov.co/sigotn/frames_pagina.aspx. Instituto Geográfico Agustín Codazzi (IGAC) (2015). Avalúos catastrales totales (urbano y rural) (data file). Retrieved from  http://sigotn.igac.gov.co/sigotn/reporteMapaNuevo.aspx?prmAlls=750;6;2015;1;0;0;655674;Aval%C3%BAos%20Catastrales%20Totales%20(Urbano%20y%20Rural); Instituto Geográfico Agustín Codazzi (IGAC) (2016). Avalúos catastrales totales (urbano y rural) (data file). Retrieved from http://sigotn.igac.gov.co/sigotn/reporteMapaNuevo.aspx?prmAlls=750;6;2016;1;0;0;655674;Aval%C3%BAos%20Catastrales%20Totales%20(Urbano%20y%20Rural); Data from the National Department of Planning (Departamento Nacional de Planeación) of Colombia (2014) provides the “debt”, “savings”, “resources”, and “investment” variables for the municipal government (2015a, b, c). Departamento Nacional de Planeación. (2014). *Magnitud de la deuda municipal* (data file). Retrieved from: http://sigotn.igac.gov.co/sigotn/reporteMapaNuevo.aspx?prmAlls=223;3;2014;1;0;0;655674;Magnitud%20de%20la%20Deuda%20Municipal; Departamento Nacional de Planeación. (2015a). *Capacidad de ahorro* (data file). Retrieved from: http://sigotn.igac.gov.co/sigotn/reporteMapaNuevo.aspx?prmAlls=209;3;2015;1;0;0;655674;Capacidad%20de%20Ahorro; Departamento Nacional de Planeación. (2015b). *Generación de Recursos Propios (Esfuerzo Fiscal)* (data file). Retrieved from: http://sigotn.igac.gov.co/sigotn/reporteMapaNuevo.aspx?prmAlls=87;3;2015;1;0;0;655674;Generaci%C3%B3n%20de%20Recursos%20Propios%20(Esfuerzo%20Fiscal); Departamento Nacional de Planeación. (2015c). *Magnitud de la inversión municipal* (data file). Retrieved from: http://sigotn.igac.gov.co/sigotn/reporteMapaNuevo.aspx?prmAlls=212;3;2015;1;0;0;655674;Magnitud%20de%20la%20Inversi%C3%B3n%20Municipal; Data on Population density was downloaded from the Departamento Administrativo Nacional de Estadística (DANE). Departamento Administrativo Nacional de Estadística (DANE) (2018). Archivo de estimación y proyección de población nacional, departamental y municipal por área 1985-220 (for years 2015–2017) (data file). Retrieved from http://www.dane.gov.co/reloj/.

## Abbreviations

ZikaDens	The number of reported cases of Zika virus infection divided by the weighted projected population density from the same time period. This is the number of cases per people per square kilometer (cases/people/km^2^)
ZikaSize	The number of reported cases of Zika virus infection multiplied by 10,000 and divided by the weighted projected population size. This would be the number of cases per 10,000 people of the population (cases/10,000)
ENSO	El Niño-Southern Oscillation
DANE	National Administrative Department of Statistics (Departamento Administrativo Nacional de Estadística
IGAC	Geographic Institute Agustín Codazzi (Instituto Geográfico Agustín Codazzi)
INS	National Institute of Health (Instituto Nacional de Salud)

## Appendix A

Article 102 of Colombian “Law 142 of 1994” (Law 142 of 1994 (11 July), 1995) states [68]: “Residential properties to which public services are provided will be classified in six socioeconomic strata as follows: (1) low-low, (2) low, (3) medium-low, (4) medium, (5) medium-high, and 6) high” (p. 51). Article 102 also says: “No urban residential area that lacks the provision of at least two basic public residential services may be classified in a stratum higher than four” (Law 142 of 1994 (11 July), 1995, p. 51). Thus, it appears that lacking basic residential services is a sign of lower socioeconomic status. It seems that residential properties that are classified as being in strata 1, 2, or 3 would be in lower socioeconomic strata than residential properties that are classified as being in strata 4, 5, or 6.

This law indicates that users within some strata can receive subsidies. Article 87.3 of this law states: “Solidarity and redistribution means that in implementing the tariff system, measures will be taken to allocate resources to ‘solidarity and redistribution funds’ so that users of the upper strata and commercial and industrial users can help users of low strata to pay the rates of services that cover their basic needs” (Law 142 de 1994 (11 July), 1995, p. 43). Article 89 states (based on a Google translation, with emphasis added): “The regulatory commissions will gradually require all those who provide public services that, in collecting the tariffs that are in force upon enactment of this Law, distinguish on the invoices between the value that corresponds to the service and the factor that is applied, to give subsidies to the users of strata 1 and 2. They will also define *the conditions for applying [subsidies] to stratum 3*” (Law 142 of 1994 (11 July), 1995, p. 44, emphasis added). Article 89 goes on to state: “The resources of said funds [solidarity funds] will be destined to give subsidies to users of strata 1, 2 and 3, as social investment, under the terms of this Law” (Law 142 of 1994 (11 July), 1995, p. 44). Article 99.6 states (with emphasis added):

The part of the fee that reflects the costs of administration, operation, and maintenance to which the supply results will always be covered by the user; the part of the fee that has the purpose of recovering the value of the investments made to provide the service may be covered by the subsidies, and provided they are not, the utility can take all the necessary measures for users to cover them. In no case shall the subsidy exceed 15% of the average cost of supply for tier 3, or 40% of the average cost of supply for tier 2 or more than 50% of tier 1 cost for tier 1. Subsidies will only be granted to users of residential properties and to *rural areas of strata 1 and 2; the regulatory commissions will define the conditions for granting them to stratum 3* (Law 142 of 1994 (11 July), 1995, p. 49, emphasis added).

Therefore, it appears that subsidies are given to residential properties classified in strata 1 and 2, and possibly to those in strata 3. However, there are more articles to the law that we did not read. As such, it is possible that not all residential properties that are classified in strata 1–3 receive subsidies. Based on the categorical classification of the strata representing “(1) low-low, (2) low, (3) medium-low, (4) medium, (5) medium-high, and (6) high” socioeconomic strata ((Ley 142 de 1994 (julio 11), 1995, p. 51) it could be that grouping them into strata 1–2, 3–4, and 5–6 is more appropriate. Although, restricting the “Energy A” variable to just those that contained individuals in strata 1 and 2 could change the results, however, we would not expect this to dramatically change our results.
